# Supplementary material for: BioCRNpyler: Compiling chemical reaction networks from biomolecular parts in diverse contexts
Source: PLoS Comput Biol. 2022 Apr 20;18(4):e1009987. doi: 10.1371/journal.pcbi.1009987 (PMC9060376; doi:10.1371/journal.pcbi.1009987)
Supplement: Attachment — Submitted filename: BioCRNpyler_reviewer_responses.pdf [file pcbi.1009987.s002.pdf]

In the following document, we have reproduced the reviewer comments and addressed them in blue. In the process of these revisions, we have made substantial improvements to the BioCRNpyler manuscript including:

- Restructuring the paper so that details of the compilation algorithm come before the example models and numerous textual changes to make this restructuring cohesive.
- A more detailed comparison of BioCRNpyler to other related pieces of software, including an overview table in the Discussion section highlighting similarities and differences.
    - This text and table are included in our responses to reviewer 1 comment 2.
- Two additional subsections: 1.7 and 1.8 which provide code snippets of a BioCRNpyler specification and a step-by-step description of the compilation algorithm, respectively.
    - We have included section 1.7 in our response to reviewer 1 comment 1 and figure 3 (which goes along with section 1.8) in our response to reviewer 3 comment 4.
- Additions to figure 3 (formerly 2) to include an overview of the compilation algorithm.
- Additions to figure 5 (formerly 3) showing simulation details of the Lac Operon Model.
- A simplification of the models in figure 6 (formerly figure 5) to more clearly explain component enumeration.
- References to supplemental tables and supplemental sections in the primary text which detail the biocrnpyler library and describe how to expand it.
- Additional supplemental information sections giving detailed descriptions (and additional code) of how all the different classes and objects used to produce the example models work.
- An index (on Github) showing where to find detailed examples for different BioCRNpyler classes.

We believe these changes have dramatically improved the BioCRNpyler manuscript and thank the reviewers for their helpful feedback on this work.

Reviewer #1:

The paper presents a new compiler from genetic circuit designs specified in a high-level language into chemical reaction network models appropriate for simulation and other analyses. This high-level language and compiler can greatly reduce the size of the description of practical genetic circuit designs as demonstrated in their results shown in Figure 1G. Therefore, this tool is likely to prove to be very useful. The authors are commended for providing this open source with detailed documentation, examples, and tutorials.

The paper though needs some work to better articulate their contributions while positioning themselves with respect to the related work. Here are my more detailed comments:

1) Abstract (and introduction) - you talk about the high-level design specification, which is your key contribution, but very little detail about it is provided in the abstract or introduction. It would be useful to provide a bit of a description in both the abstract and introduction, so the reader can understand at what level of abstraction it works and provide some intuition why it may result in reductions in model complexity. A small motivating example early in the paper would likely be useful to engage the reader sooner. Indeed, all the examples of the actual specification seem to only appear in the supplemental.

Thank you for this helpful comment. We have added text to both the introduction and abstract to begin explaining how BioCRNpyler works at a high level. We have also re-organized the paper so that details of the software compilation algorithm are explained before the motivating examples in order to better use the examples as a way of illustrating the novelty of our approach. In the initial explanation, we also focus on a simple model of transcription and translation of a single gene in order to clarify the discussion with an example (including code snippets). Specifically (captured as a screen snip):

1.7 Specification Example

Before describing the compilation algorithm in detail, we illustrate the central idea of a BioCRNpyler specification via an example involving a DNAassembly Component which represents a simple piece of DNA called X with a promoter (prom), ribosome binding site (rbs), and coding sequence for a protein. The DNAassembly uses transcription and translation Mechanisms which will be placed into a Mixture.

```
# Create Mechanisms
tx = SimpleTranscription() #Transcription
tl = SimpleTranslation() #Translation
# Create a Component
G = DNAassembly("X", promoter = "prom", rbs = "rbs", protein = "X")
# Define Parameters
params = {"kb":100, "ku":10, "ktx":0.1, "ktl":0.5,}
# Place the Component and Mechanisms in a Mixture
M = Mixture("mixture", components = [G], mechanisms = [tx, tl], parameters = params)
# Compile the CRN
CRN = M.compile_crn()
```

This simple code compiles the CRN:

$$X_{\mathrm{DNA}} \xrightarrow{0.1} X_{\mathrm{DNA}} + X_{\mathrm{RNA}} \qquad X_{\mathrm{RNA}} \xrightarrow{0.5} X_{\mathrm{RNA}} + X_{\mathrm{Protein}}. \tag{1}$$

The modularity of BioCRNpyler can be illustrated by considering what would happen if we instead used “Michaelis Menten” transcription and translation Mechanisms which model RNA-polymerase (P) and ribosomes (R):

```
tx = Transcription_MM(rnap = Species("P")) #Transcription Mechanism
tl = Translation_MM(ribosome = Species("R")) #Translation Mechanism
```

This compiles a considerably more complex CRN:

$$X_{\mathrm{DNA}} + P \underset{10}{\overset{100}{\rightleftharpoons}} X_{\mathrm{DNA}}{:}P \xrightarrow{0.1} X_{\mathrm{DNA}} + P + X_{\mathrm{RNA}}$$
$$X_{\mathrm{RNA}} + R \underset{10}{\overset{100}{\rightleftharpoons}} X_{\mathrm{RNA}}{:}R \xrightarrow{0.5} X_{\mathrm{RNA}} + R + X_{\mathrm{Protein}}.$$

Here, “:” indicates that two species are bound together to form a new species.

2) Intro - "relatively few tools exist to aid in the automated construction of general CRN models from simple specifications", I'm not so sure I completely agree with this statement. There are several tools that the authors do not reference that do just this, such as Antimony, ShortBOL, and the Virtual Parts Repository. Even pySBOL coupled with model generation tools such as VPR or iBioSim provide similar functionality to what they are proposing. I do believe that their approach is novel and useful, but the authors need to do a better job articulating the similarities and differences with these other approaches.

We have added clarifications to the introduction in order to more comprehensively describe other software tools (including removing the misleading words “relatively few”). Additionally, we have added a detailed comparison of BioCRNpyler to other tools in the discussion section, including a table highlighting some of the key similarities and differences. Specifically, in the introduction we have written:

> “There are many existing tools that provide some of the features present in BioCRNpyler. Antimony (part of the Tellerium software suite) provides an elegant highlevel language that is converted into SBML models [12, 30]. Systems Biology Open Language (SBOL) [31] is a format for sharing DNA-sequences with assigned functions and does not compile a CRN. Hierarchical SBML and supporting software provide [32] provides a file format which encapsulates CRNs as modular functions. The software package iBioSim

[33, 34] can compile SBOL specifications into SBML models. Similarly, Virtual Parts Repository uses SBOL specifications to combine existing SBML models together [35]. The rule-based modeling framework BioNetGen [36] allows for a system to be defined via interaction rules which can then be simulated directly or compiled into a CRN. Similarly, PySB [37] provides a library of common biological parts and interactions that compile into more complex rule-based models. Finally, the MATLAB TX-TL Toolbox [38, 39] can be seen as a prototype for BioCRNpyler but lacks the object-oriented framework and extendability beyond cell-free extract systems.

BioCRNpyler compliments existing software packages by providing a novel abstraction and framework which allows for complex CRNs to be easily generated and explored via the compilation process. To do this, BioCRNpyler specifies a biochemical system as a set of modular biological parts called Components, biochemical processes codified as CRNs called Mechanisms, and biochemical and modeling context called the Mixture Moreover, BioCRNpyler contains an extensive library of Components, Mechanisms, and Mixtures allowing for synthetic biological parts and systems biology motifs to be reused and recombined in diverse biochemical contexts at customizable levels of model complexity with minimal coding requirements (BioCRNpyler is designed to be a scripting language). Additionally, BioCRNpyler is purposefully suited to in silico workflows because it is an extendable object-oriented framework written entirely in Python that integrates existing software development standards and allows complete control over model compilation. Simultaneously, BioCRNpyler accelerates model construction with extensive libraries of biochemical parts, models, and examples relevant to synthetic biologists, bio-engineers, and systems biologists."

And in the discussion we have written:

"Given the plethora of model building and simulation software already in existence, it is important to highlight how BioCRNpyler fits into the larger context of existing tools. Table 1 gives a high level overview of how BioCRNpyler compares to other tools. Firstly, BioCRNpyler stands out due to the novel Mixture-Component-Mechanism abstraction. This framework allows users to easily put together complex models using BioCRNpyler's extensive library or to develop their own extensions by writing Python code. Rule based frameworks, such as BioNetGen [36] and PySB [37] offer similar abstractions to Mechanisms. However, these must be codified in a formal language specific to the framework (BioNetGen uses .bng files and PySB uses a specialized text format) which offers less flexibility than the arbitrary python code allowed by BioCRNpyler. The Virtual Parts Repository [35] and iBioSim [34] take a different approach to abstract specifications by generating CRNs from SBOL files. This methodology is similar in spirit to BioCRNpyler but is restricted due to the reliance on the SBOL standard, the need of software-specific SBOL annotations, and challenges in generalizing beyond gene regulatory network architectures. BioCRNpyler also differs from many other pieces of software because it includes a detailed library of biological parts and models. PySB, Virtual Parts Repository, and iBioSim similarly include a variety of built-in rules, models, and parts, respectively. However, BioCRNpyler is unique in its modularity: the ability to

use the same Component with different Mechanisms placed in different Mixtures allows for a combinatoric variety of models to be easily specified and explored. Finally, we reiterate that BioCRNpyler is not a CRN simulator like COPASI [11], MATLAB Simbiology [13], or Tellurium (via libroadrunner) [12, 14]. This brings us to a final point about BioCRNpyler: it is a pure Python package with very minimal dependencies meant to be used as a scripting language, interfaced with existing simulators, used in Jupyter notebooks [67], and integrated into existing pipelines."

And included the following comparison table:

Software	Abstraction	Library	Simulator	Source	UI	API
**BioCRNpyler** [40	`Mixtures`, `Components`, `Mechanisms`, & CRNs	Yes	No	Python	Python	Python
**BioNetGen** [36]	Rules	No	Yes	Perl C++ Python	.bng files	.bng files
**PySB** [37]	Rules	Yes	No	Python	Text Rules	Python
**Tellurium** [12] (using Antimony [30] and libRoadrunner [14])	CRNs	No	Yes	Python	Text Reactions	Python
**Virtual Parts Repository** [35]	SBOL	Yes	No	Java	Web	Java
**iBioSim** [34]	SBOL & CRNs	Yes	Yes	Java	GUI	Command line
**COPASI** [11]	CRNs	No	Yes	Java C++	GUI	C++ & other derived APIs
**MATLAB Simbiology** [13]	CRNs	No	Yes	MATLAB	MATLAB	MATLAB

Table 1. Comparison of different simulation software. **Abstraction:** how models can be represented in the software. **Library:** whether there is a substantial library of pre-existing parts/components/sub-models that can be reused. **Simulator:** whether the software simulates models numerically. **Source:** the language(s) the software is written in. **UI:** the primary way a user interacts with the software. **API:** the primary programming language the software is designed to be accessed with.

3) Page 8 and a few other places, "objected-oriented" -> "object-oriented"

These typos have been fixed.

4) Figure 1G is the key result demonstrating the utility of this work. A bit more intuition for this result is needed. Part of the issue is not having a detailed example up to this point.

We hope that by moving Figure 1 to after a description of how compilation works (it is now Figure 4) better enables the reader to understand how this result is possible. Additionally, in the

newly written compilation section we have added an even simpler example (including code - see above) to better illustrate how the software works concretely.

5) Figure 6, repeated "relatively" in the caption.

This typo has been fixed.

6) A key contribution cited is the flexibility to have alternative models. My understanding is this requires writing python code to create new mechanisms. In some sense, this lessens the impact of this contribution, since other model generators can (and do) provide alternative modeling methods via added code to their model generators. It is unclear to me how much easier it is to develop a new model using their approach versus other model generators. A detailed example of how this is done would be useful to demonstrate this.

We understand the concern that subclassing Mechanisms (and other objects) requires substantial coding experience. For this reason, we have spent considerable effort building up BioCRNpyler as a library of many Mixtures, Mechanisms, and Components which can be mixed and matched to create a huge variety of models. We have attempted to emphasize this point in the text in a variety of places and have added explicit references to supplemental tables detailing many of the existing parts of the library in the supplement. We have also explicitly referenced supplemental sections (which were already in the previous draft, but unreferenced in the text) detailing how to subclass key BioCRNpyler objects. Finally, we note that we have an example notebook showing how to subclass the core BioCRNpyler objects which has been successfully used by non-CS undergraduates (e.g. bioengineers) to build their own custom classes.

Overall, this is a useful tool that may have the potential to enable model-driven design for biologists and bioengineers with limited programming experience. The authors need to be clear that some experience is still needed though, as python programs still need to be created. The authors need to also better articulate the differences between their tool and similar approaches.

We have emphasized in the abstract and introduction that BioCRNpyler is designed as a scripting language in order to clarify the level of programming experience needed to use the package. Additionally, as mentioned above, we have expanded our comparison to other tools in both the introduction and discussion.

Reviewer #2:

The manuscript describes a Python package for creating of reaction networks in SBML from a high-level design specification. It will be very useful for biologists using Python for modeling.

Pros:

- Easy to install, no extra libraries were required on Mac. A lot of tutorials and examples.

- SBML is correct, tested by importing into COPASI general simulator. Simulations run nicely with LibRoadRunner from Jupyter notebook.

- The libraries of components and mechanisms are useful for compiling multiple models from standard parts.

- Creating new components and mechanisms is very useful way to extend these libraries.

- A very interesting and useful approach of maintaining a parameter database with hierarchy of components and mechanisms (types have multiple names) and automatic substitution of parameters using this hierarchy, for example if no parameter exists for a specific mechanism name, but a parameter value exists for a mechanism type.

Major issues:

1. The manuscript is rather difficult to read. It’s neither a biological paper describing a use of a modeling approach, nor a computational paper describing a software.

> a. The manuscript lists a lot of Python classes but does not give any details on how they work. The only way to understand how the package works is to follow examples on GitHub and run them one by one. For example, the authors mention OrderedPolymerSpecies and a PolymerConformation, but never explain what are these, or how DNAconstruct enumerates parts. Most classes are defined so briefly that it’s impossible to understand what they do. “GlobalMechanisms are rules used to generate Species and Reactions at the end of compilation” – it is repeated three times in different contexts, but it does not help with understanding of how it works. …DillutionMixture is neither defined biologically nor explained programmatically, despite being used many times in different contexts. I just recommend the authors to look for any complicated Python class name in the manuscript and ask themselves whether the description in the manuscript is enough to understand the term.
>
> This is great advice to make the paper more readable. We have attempted to remove most references to particular pieces of code from the text unless we discuss that object extensively (such as for Components, Mechanisms, and Mixtures). Additionally, we have

added detailed descriptions of advanced classes used in the example models we describe to the supplemental material for an interested reader.

b. The use of the term “hierarchical” and mentioning SBML hierarchical extension are misleading: BioCRNpyler is not generating hierarchical models.

We agree - BioCRNpyler does not produce hierarchical SBML models at this point. However, the organization of BioCRNpyler is hierarchical. In order to avoid confusion we have confirmed that the word “hierarchical” is only used once outside the context of discussing Hierarchical SBML. In this case, “hierarchical” is clearly describing the class structure of BioCRNpyler and SBML is not mentioned at all to avoid confusion.

c. There are many GitHub folders with many tutorials. Some guide on which classes are described in which tutorials would be helpful.

This is an excellent suggestion. We have created an index “0. Tutorial Index.txt” in the examples folder which shows which tutorial jupyter notebook different BioCRNpyler classes first show up in (this is done via a script so the index can be updated as the tutorials expand).

2. The authors mention several tools that are comparable to BioCRNpyler, but don’t compare and don’t demonstrate current (not potential) advantages over other tools.

a. At which point the use of BioCRNpyler becomes easier than specifying models in SBML simulators like Copasi and Tellurium? The complex model of Lac Operon is definitely easier to define in any rule-based language, but is BioCRNpyler better than BioNetGen or PySB?

We do not believe either framework is “better” – they are simply different. Rule based models are formal and require a very specific semantics; some users might find such structure to be more clear and rigorous. BioCRNpyler is designed to be less constrained via the ability to implement functionality directly with Python code; our experience is that many users (especially python scripters and developers) find this to be more intuitive than learning to use a rule based framework.

b. What’s the difference with PySB? I noticed parameters, but otherwise the same mechanisms can be defined in PySB, and using BioNetGen in PySB is more powerful.

BioCRNpyler is different from PySB and BioNetGen and we do not claim to be more powerful. We have added a more detailed comparison of this point in the Discussion and the Introduction sections (you can see excerpts of this text in the response to reviewer 1, comment 2) and also respond to some of your specific questions below.

c. What rule-based features are used? Is it a plain combination of all components without any constraints?

BioCRNpyler is not a rule based framework, in the formal sense. However, species and reactions can in theory be generated via arbitrary python code during the compilation process so any programmable constraint is, theoretically, implementable within the BioCRNpyler framework. In particular, if Components are somehow designed to interact with other Components, the Component Enumeration step allows for compile-time generation and modification of BioCRNpyler objects before species and reactions are enumerated.

d. More comparing with BioNetGen would be useful – are there any advantages of BioCRNpyler high-level specification over the BNGL language? Can it specify something that BioNetGen cannot?

Towards this end, we have added a more detailed discussion of BioCRNpyler and other frameworks (including PySB and BioNetGen) to the discussion section and a table highlighting some of the key similarities and differences.

Responding to your question here: although we are not experts in BioNetGen or rule based modeling in general, our view is that these packages should be about equal to BioCRNpyler in terms of expressivity. Formally, both systems are able to enumerate arbitrary and arbitrarily large chemical reaction networks. We note that using BioNetGen or Kappa to simulate “infinite CRNs” is something BioCRNpyler cannot do because it is not a simulator, it simply produces finite SBML models. From a practical point of view, rule-based modeling provides a rigorous formal semantics for reaction enumeration whereas BioCRNpyler provides a compilation methodology and replaces formal semantics for more or less arbitrary python code. From this perspective, each framework has advantages and disadvantages. Rule-based models are more formal than typical BioCRNpyler models. However, many modelers (including ourselves) find rule-based modeling to be difficult and prefer to work in more familiar programming languages. In this sense, we see BioCRNpyler as giving us the expressivity of an abstract rule-based language with freedom to write whatever code we want. Indeed, one could even imagine using pySB or BioNetGen inside of a BioCRNpyler class in order to perform reaction enumeration. This is clearly the perspective of a very advanced modeler. We believe that from a simple scripting methodology, BioCRNpyler is quite easy to use and has been tested by numerous biologists and bioengineers at various levels of programming literacy.

e. Comparing with iBioSim would be helpful. It also can define genetic components and mechanisms.

We are unsure what specific iBioSim feature you are referring to. That said, we have added explicit references to iBioSim’s ability to generate gene regulatory network models from SBOL specifications to the discussion section of the paper. To the best of our knowledge, the specific models used for these networks are fairly static and not programmatically accessible without modifying iBioSim’s source code. We also

emphasize that one reason for developing BioCRNpyler was to have a python based tool to add to python workflows whereas iBioSim is written in Java.

f. Comparing with Tellurium/Antimony would be helpful – it has human-readable language.

We have added more careful references to Tellurium/Antimony in both the introduction and discussion. Indeed, we believe that integrating something like BioCRNpyler into the Tellurium environment could be very powerful (we already have integrated BioCRNpyler with libroadrunner).

g. The manuscript will gain a lot if an example will be provided (may be as a supplement) in all four languages: BioCRNpyler, PySB, BioNetGen, and Antimony.

Although we appreciate this reviewer's interest in more detailed comparisons between these different software packages, we do not feel that such a comparison is fair for a variety of reasons. Firstly, for very simple CRN models which can be written by hand, rule-based models (and BioCRNpyler using compilation) will likely seem much more complicated than just writing out the CRN in Antimony or directly using BioCRNpyler (without compilation). On the other hand, describing complex models which require significant compilation/enumeration is difficult to do fairly because finding the shortest (or clearest) program (in biocrnpyler, PySB, or BioNetGen) to generate a given model is not obvious, especially considering that we are not experts at using all of these tools. Instead, we have tried to better articulate the differences in how these different pieces of software specify systems abstractly in a new table in the discussion section and accompanying text (which we have also reproduced in our response to reviewer 1, comment 2).

3. The only biological use of BioCRNpyler is in Ref 56, but it is not discussed in the manuscript, all examples are just test examples that repeat well-known and many times modelled biological systems that are simple to be defined in any biomodelling simulator.

We have expanded our discussion of pieces of software which reference Biocrnpyler to (briefly) cover some of the kinds of models which it has been used to produce. Specifically, we wrote:

> "BioCRNpyler has already been deployed to build diverse models in synthetic biology including modeling bacterial gene regulatory networks [64], modeling bacterial circuits in the gut microbiome [65], and modeling cell extract metabolism [66]."

We also believe that the Lac Operon model and integrase models are not that easily defined directly as CRNs and have expanded on how these models work in the results section and supplemental information sections. Moreover, we emphasize that the key use-case of BioCRNpyler is not just generating large models - it is providing a systematic computational methodology to change modeling assumptions and abstracts without having to redefine an entire hand-written model. For example, the exploration of a Repressillator model with multiple ribosome occupancy described in Figure 4F would both be laborious to write by hand (without

substantial coding) and also illustrates the ability of BioCRNpyler to take the same specification of a Repressillator circuit and examine its behavior under different modeling assumptions very efficiently.

4. Lac Operon model is the most complicated model described, but the authors don't mention what to do with their model of 173 species and 343 reactions, is it comparable with any previous models? And then, I could not find the code for this specific model among examples.

We have added simulation results to the Lac Operon figure showing the output of the model. We note that this model gives rise to a lag between depletion of glucose and steady-state lactose metabolism, as commonly referenced in experimental and computational studies of the lac operon. Additionally, we have added code for this example to the supplemental information and described how the core classes used in this model work. We will also upload this code (and code for all the other examples in the paper) to our Github examples.

Minor issues:

1. Biorxiv 50, 55-57 – provide full citations.

2. Ref 54 is just a review mentioning BioCRNpyler, it does not demonstrate that "BioCRNpyler has already been deployed to build diverse models in systems and synthetic biology.

3. Ref 55 is not using BioCRNpyler.

Responding to Minor issues 1-3. Sorry for these errors. We have updated the references and expanded on software which uses BioCRNpyler to be more clear and accurate (see the text included in the response to major issue 3 of this review).

4. Why the new term "mixture" is introduced instead of a classical "model"?

This is a good question. We use the term Mixture (instead of model) to differentiate the compiled CRN model from the Context (Mixture) of the BioCRNpyler abstract representation. We have attempted to clarify what Mixture means throughout the text - namely that it is BioCRNpyler's terminology for context - beginning in the introduction and expanded on in later sections.

Reviewer #3:

This work by Poole et al. introduces BioCRNpyler as a tool for users to build reaction networks from high-level design specifications. The tool seems to automate several steps of the network-building process and provides a library of biochemical reactions to build networks using these components. The user is also offered a library of parameters which provides a good starting point for simulation.

Overall, I really wanted to like this work but I feel the authors missed a chance to present their work and build enthusiasm for their tool. The paper itself is structured in a way that makes it hard to understand what and how the tool works and what the benefit of the tool would be for a reader. Rather the paper reads like a user manual with many examples/tutorials rather than a narrative about the tool.

Major comments:

1. The introduction provides a good context for how CRNs are used but it does not provide a compelling argument for why BioCRNpyler is needed. Why is compiling reactions better than what other tools do? Why would the end-user pick BioCRNpyler over other tools? I think a brief introduction to molecular compilers and their use in DNA circuits could help place the tool in context for the reader.

Thank you for this helpful comment. In the introduction, we have provided more background on molecular compilers in the context of DNA circuits and used that to motivate BioCRNpyler as a software package. In particular about DNA compilers we have written:

> "This package is inspired by the molecular compilers developed by the DNA-strand displacement community and molecular programming communities which, broadly speaking, aim to compile models of DNA circuit implementations from simpler CRN specifications [20–22], rudimentary programming languages [23, 24], and abstract sequence specifications [25]. This body of work has demonstrated the utility of molecular circuit compilers and highlights that a single specification can be compiled into multiple molecular implementations which in turn can correspond to multiple CRN models at various levels of detail. For example, there are multiple DNA-strand implementations of catalysis [21, 22, 26, 27] and the interactions of the DNA strands involved in each of these implementations can be enumerated to generate different CRN models based upon the assumptions underlying enumeration algorithm [28]. Drawing from these inspirations, BioCRNpyler is a general-purpose CRN compiler capable of converting abstract specifications of biomolecular components into CRN models with full programmatic control over the compilation process."

2. In the introduction, the authors mention what existing tools are comparable to BioCRNpyler. I think that a table in the results section would be better suited to provide this information, along with some benchmarks in the supplement.

This is also a helpful suggestion. We have clarified the capabilities of other existing tools in the introduction and compared these capabilities to BioCRNpyler in the discussion including a new table in the discussion section highlighting some of the key differences. You can see excerpts of this text in the response to reviewer 1, comment 2.

3. The authors provide a "laundry list" of motivating examples to describe BioCRNpyler. However, these examples are hard to follow. First, the biology context is not very familiar for most readers. Second, Each section is one or two paragraphs with a large amount of data/figures, making it hard to follow for readers. Third, the authors reference software calls without context. This all makes it very hard to follow.

We have re-organized the paper to present the details of how the software compilation works first and then included the motivating examples at the end. Furthermore, we have rewritten how the motivating examples are introduced so they better illustrate how to use the BioCRNpyler framework and library. Finally, we have removed most references to software calls and classes except for the central classes Component, Mechanism, and Mixture which we expand on in depth.

4. Figure 2 is not very informative. It is meant to provide a hierarchical organization of BioCRNpyler but it left me feeling lost. What am I supposed to learn from this figure? Perhaps the author should consider replacing this with a flowchart.

We have added a step-by-step description of how compilation works to this figure (now Figure 3 in the revised draft). We have copied an image of the new figure below which breaks the compilation process into 7 steps which we have referenced and elaborated in extensively in a new section ‘1.8 Chemical Reaction Network Compilation”.

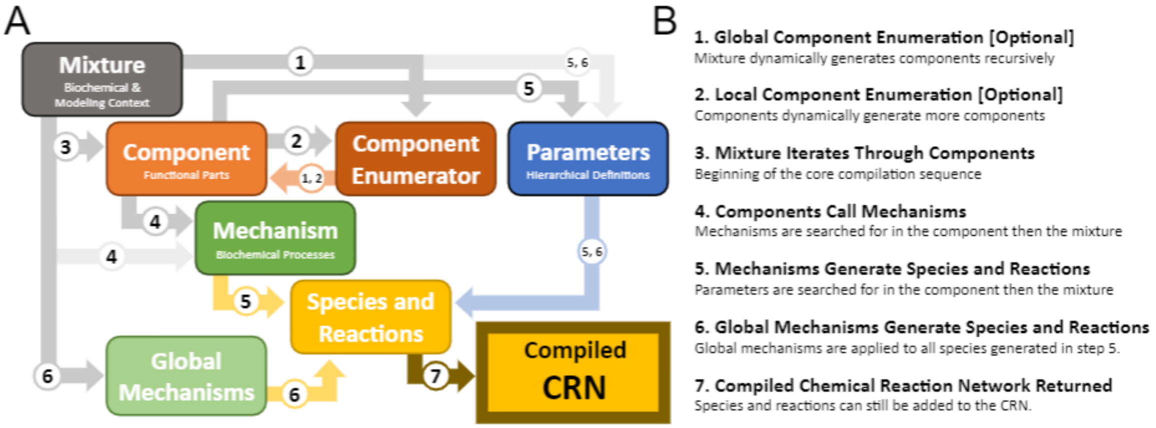

Figure 3. A. the organization of classes in BioCRNpyler. Gray arrows indicate the hierarchical organization of objects (e.g. `Components` are contained in a `Mixture`). Dark gray arrows take precedence over light gray arrows (e.g. a `Component` will search for `Mechanisms` in itself before looking at its `Mixture`). Colored arrows denote the generate of objects: `Components` are orange, parameters are blue, and CRN species and reactions are yellow. B. The compilation sequence in BioCRNpyler. The numbers on the arrows in (A) indicate which part of compilation these connections are involved in.

5. I think the readers of this journal would mostly benefit from a well-explained example throughout. This could be perhaps the Lac Operon model from Figure 3. Using one example may better help readers understand the tool.

Thank you for this helpful suggestion. We have attempted to reference transcription of a gene repeatedly throughout section one (Framework and Compilation Overview) when describing the BioCRNpyler abstraction in order to give the reader a simple example. Furthermore, we have added a new section '1.7 Specification Example' which describes a BioCRNpyler specification for a very simple gene along with code snippets. This section has been included in our response to reviewer 1, comment 1. Finally, we have moved the Lac Operon model to section 2 (results) as more complex example illustrating the utility of the package after the basics have already been explained.

6. The idea of reaction schemas seems very interesting/compelling for this work! I would like to see this idea expanded and explained in a biological context more thoroughly. The authors instead start by defining the need for a schema but quickly devolve into mathematical and algorithmic details that likely belong in the supplement or in a more specialized section.

You are indeed correct that reaction schemas are at the heart of why BioCRNpyler works and we appreciate the suggestion to add some biological context to help explain the idea. Towards this end, we have added the following text to the Mechanism section (1.2) to help clarify the biological significance of our methodology. Specifically, we have written:

"Reaction schemas refer to BioCRNpyler's generalization of switching between different mechanistic models: a single process can be modeled using multiple underlying motifs to generate a class of models which may have qualitatively different behavior. Mechanisms are the BioCRNpyler objects responsible for defining reaction schemas. In other words, various levels of abstractions and model reductions can all be represented easily by using built-in and custom Mechanisms in BioCRNpyler. Biologically, reaction schemas can represent different underlying biochemical mechanisms or modeling assumptions and simplifications. For example, to model the process of transcription (as shown in Figure 1), BioCRNpyler allows the use of various phenomenological and mass-action kinetic models by simply changing the choice of reaction schema. The simplest of these schemas "Simple Transcription" includes no details about how a gene produces a transcription. "Michaelis Menten Transcription" elaborates on this simplification by including the RNA polymerase enzyme in the model. "Michaelis Menten Transcription with a Hill Function" simplifies the previous mass action model assuming a quasi-equilibrium approximation of RNA polymerase binding. Finally, the "Multi-Occupancy Michaelis Menten Transcription Model" aims to be more realistic by examining the possibility of multiple RNA polymerase enzymes bound to a single transcript. Of course, these are not the only possible transcription Mechanisms: more detailed models may include transcript elongation or organism-specific co-factors, such as σ-factors in E. coli, which could also easily be included in a BioCRNpyler Mechanism."

Minor comments:

1. Various spelling mistakes are present throuhgout. For example, in the abstract the authors wrote "complies" when I believe they meant "compile".

We have attempted to fix as many typos as we could.

2. Similarly, the tone of the paper often sounds like a user manual or an advertisement for BioCRNpyler rather than a tool that solves a biological problem.

We hope that by reorganizing the paper to focus more on the underlying algorithm and abstraction and how these can be applied to examples that our tone better matches what the reviewer was expecting.

3. Figure 1 is too cluttered, while Figure 2 is not very informative. It is unclear to me what the other figures are trying to convey as well. The figures work best when they flow with the narrative.

We have added considerable detail to Figure 2 (now Figure 3) in order to make it more useful. We hope that figure 1 (now figure 4) is more understandable because it follows a detailed description of the software and some simpler examples.

4. Although the authors do a decent job in the introduction to present other tools, they also miss a chance to place BioCRNpyler in the context of other python tools. For example, Tellurium, COBRApy, COPASI, etc are Python tools that may be complementary to BioCRNpyler and should at least be mentioned.

We have explicitly referenced these tools in the discussion at the end of the paper, including a comparison table to better highlight the similarities and differences from BioCRNpyler. We have also reproduced pieces of this text discussing BioCRNpyler in relation to other modeling software in our response to reviewer 1, comment 2.
