# Supplementary material for: BioCRNpyler: Compiling chemical reaction networks from biomolecular parts in diverse contexts
Source: PLoS Comput Biol. 2022 Apr 20;18(4):e1009987. doi: 10.1371/journal.pcbi.1009987 (PMC9060376; doi:10.1371/journal.pcbi.1009987)
Supplement: Attachment — Submitted filename: Reviewer Resposnes 2.pdf [file pcbi.1009987.s003.pdf]

BioCRNpyler Reviewer Responses 2nd Revision

Responses to reviewers are indicated in blue.

Comments to the Authors:
Please note here if the review is uploaded as an attachment.

Reviewer #1: The authors have addressed all my concerns. We are pleased that we have adequately addressed all your concerns.

Reviewer #2: The authors significantly improved the readability of the manuscript. Thanks! We also are very happy with how the first round of reviewer comments and revisions greatly improved the manuscript.

My general note is about supplemental material. In Figures 4 and 6 it would be very useful to quickly look into the supplemental material and find a snippet of a code describing multiple models mentioned in the manuscript. However, there are no direct references to supplemental material describing specific code. The same way as the authors refer to specific supplemental tables, it would be nice to refer to specific sections of supplemental material describing models. Now a reader needs to read the whole supplemental material to match it to specific figures in the manuscript. We have added specific references to specific supplemental information containing code for the different example models.

There are minor issues in the manuscript that should be fixed: Thank you for pointing out these final issues. We have attempted to fix all of them as described below.

Lines 91-96 are almost verbatim repetition of lines 70-74. Perhaps the introduction can be without specific terms that would come later. We have removed reference to the object classes from the introduction in order to keep this part of the paper at a higher level with more details coming in the following methods section.
Line 175: exclamation sign is not necessary. We have removed this punctuation.
Lines 226-227: are “a promoter (prom), ribosome binding site (rbs)” necessary for the first code? Explain when and why you’ll need them, or remove – they confuse the reader. We have removed the abbreviations in the parentheses.
Line 259: fix ComponentEnuemrators We have fixed this typo.
Line 318: is DNAasembly the same as defined in line 234? These are the same class. Instead of repeating the description of DNAassembly we have now referenced the previous section.
Figure 4: explicitly refer to each part of supplemental material for code snippets for each model. We have added text clarifying which supplemental information sections contain code for these models.
Figure 5 – what do colors for LacOperon species (light blue, dark blue, bright blue and violet) mean? These colors correspond to the different polymer conformations illustrated above the CRN diagram. We have added text in the figure caption clarifying our intended meaning which we reproduced below:

> “Figure 5. A model of the lac operon compiled using BioCRNpyler specifications with 141 species and 271 reactions using ~50 lines of code. A. A Mixture contains a set of Components and Mechanisms. The Component classes used for each element of the model are shown in brackets. The colored circles show how Components correspond to compiled CRN species in panel C. B. A schematic of the lac operon and the three looped and one open conformation it can take. Each conformation contains a combinatoric number of states based upon the

> accessible binding sites: R are lac repressor binding sites; C is the activator c-CRP binding site; P is the promoter; and Z, Y, A are the three lac genes. The conformations are placed over clusters of identically colored species corresponding to that conformation in the compiled CRN. C. A graph representation of the compiled CRN. Each circle is a unique chemical species. Square boxes show how chemical species interact via reactions generated by specific Mechanisms. D. Simulated output of the model."

Lines 424-425: "bio-modeling course and bootcamps with dozens of users ranging from college freshmen and sophomores with minimal coding experience" - it reads like a grant application. I would remove it or be more specific on why these users use this software, e.g. which biological systems do they model that it makes it easier. The importance of this testing is to highlight that BioCRNpyler is approachable to many levels of programmers. We have modified the passage to emphasize this point by writing:

> "Furthermore this software has been extensively tested via 423 inclusion in bio-modeling courses and bootcamps for users ranging from college 424 freshmen and sophomores with minimal coding experience to advanced computational 425 biologists demonstrating the accessibility and flexibility of the package."

Link http://buildacell.io/BioCRNPyler from https://github.com/BuildACell/bioCRNpyler is dead. Thank you for pointing this out. We have removed these links for now and are talking to the BuildACell webpage owners to figure out how our webpage broke.

Reviewer #3: The authors have addressed all my concerns with this revision. There are still some lingering typos throughout (e.g. complies -> compiles that should be fixed. We are pleased that we have adequately addressed all your concerns. Additionally, we have reread the paper again and corrected all the typos we could find.
